# Capsule Commentary on Ashburner et al., Electronic Physician Notifications to Improve Guideline-Based Anticoagulation in Atrial Fibrillation: a Randomized Controlled Trial

**DOI:** 10.1007/s11606-018-4678-1

**Published:** 2018-10-17

**Authors:** Ioannis Mastoris, Alexander G. Mathioudakis

**Affiliations:** 1 Division of Cardiovascular Diseases, University of Kansas Medical Center, The University of Kansas Health System, Kansas City, KS USA; 2 Division of Infection, Immunity and Respiratory Medicine, The University of Manchester, Manchester, UK

While oral anticoagulation (OAC) can mitigate the five-fold increase of ischemic stroke risk that is associated with atrial fibrillation (AF), this is at the expense of an increased risk of bleeding that often discourages clinicians and patients from prescribing OAC. Different perceptions of ostensible risk for either stroke or bleeding risk between primary care physicians and cardiologists lead to different therapeutic strategies and outcomes. Patients seen by cardiologists are 40% more likely to be prescribed OAC for AF, resulting in 40% lower risk of stroke, without a significant increase in their bleeding risk.^1^ Similarly, a TREAT-AF sub-study confirms that cardiology care in AF is associated with reduction in both stroke risk and mortality, largely due to early prescription of OAC after diagnosis.^2^ Since most patients with AF are managed in primary care and as shown, are likely to receive suboptimal management, it is crucial to implement change and to tackle the significant healthcare inequalities between patients seen by a specialist or a primary care physician.

In this issue of *the Journal*, Ashburner et al. evaluated the impact of an electronic alert tool in increasing the prescription rate of OAC in patients with AF seen in primary care setting.^3^ While this study did not show any evidence that electronic alerts would be effective towards that direction, it illustrates the challenges of clinical decision making in starting OAC. It sheds light on the reasons that prompted primary care physicians to avoid OAC, which included paroxysmal AF, perceived high bleeding risk, fall risk, and patient decision.

These concerning findings reveal a significant knowledge gap among primary care physicians, who believe their decisions not to use OAC are appropriate. As a result, patients also receive incorrect information that limit their ability to make an informed decision. A previous systematic review evaluating patient values and preferences in decision making for antithrombotic therapy revealed that well-informed patients place a higher disutility on stroke than bleeding or treatment burden and this choice must be respected by clinicians.^4^ Well-designed educational interventions targeted to primary care physicians and patients are required to address this issue.
